# LiDAR-Based 3D Scans of Soil Surfaces and Furrows in Two Soil Types

**DOI:** 10.3390/s19030661

**Published:** 2019-02-06

**Authors:** Frederik F. Foldager, Johanna Maria Pedersen, Esben Haubro Skov, Alevtina Evgrafova, Ole Green

**Affiliations:** 1 Department of Engineering, Aarhus University, Inge Lehmanns Gade 10, 8000 Aarhus C, Denmark; jp@eng.au.dk; 2 Agro Intelligence ApS, Agro Food Park 13, 8200 Aarhus N, Denmark; ehs@agrointelli.com (E.H.S.); aev@agrointelli.com (A.E.); olg@agrointelli.com (O.G.)

**Keywords:** 3D soil surface, microtopography, pinboard, furrow cross-section, trailing shoe, precision agriculture, SICK

## Abstract

Soil surface measurements play an important role in the performance assessment of tillage operations and are relevant in both academic and industrial settings. Manual soil surface measurements are time-consuming and laborious, which often limits the amount of data collected. An experiment was conducted to compare two approaches for measuring and analysing the cross-sectional area and geometry of a furrow after a trailing shoe sweep. The compared approaches in this study were a manual pinboard and a Light Detection and Ranging (LiDAR) sensor. The experiments were conducted in coarse sand and loamy sand soil bins exposed to three levels of irrigation. Using the LiDAR, a system for generating 3D scans of the soil surface was obtained and a mean furrow geometry was introduced to study the geometrical variations along the furrows. A comparison of the cross-sectional area measurements by the pinboard and the LiDAR showed up to 41% difference between the two methods. The relation between irrigation and the resulting furrow area of a trailing shoe sweep was investigated using the LiDAR measurements. The furrow cross-sectional area increased by 11% and 34% under 20 mm and 40 mm irrigation compared to non-irrigated in the coarse sand experiment. In the loamy sand, the cross-sectional area increased by 17% and 15% by irrigation of 20 mm and 40 mm compared to non-irrigated measured using the LiDAR.

## 1. Introduction

A wide range of sensors are used to describe various properties in arable ecosystems. Weed detection can be performed using high precision cameras [1], soil organic carbon stocks and many soil properties can be obtained using spectroscopic techniques [2], as well as soil surface roughness can be evaluated using scanners [3] and digital cameras [4]. Soil surface roughness is an important measure in agricultural research and engineering as it affects properties such as soil water interaction [5] and the risk of soil erosion [6,7]. The soil roughness and cross-sectional shapes of furrows have been determined to develop and assess the performance of tillage tools by measuring the resulting soil disturbance of tillage operations [8,9]. Several methods exist for evaluating the cross-sectional area and geometry of a furrow such as a pinboard [10] and a chain method [11]. These methods are manually operated and thus limited to assess only a finite number of cross-sectional profiles along a furrow. Digital and automated measurements of soil surface roughness illustrated a better description of the spatial variations in both natural and agro-ecosystems [12]. The automated measurement methods also allow for on-the-go measurements of soil roughness as shown in [3] for site-specific cultivation using a Light Detection and Ranging (LiDAR) sensor. The LiDAR has the advantage of being a non-contact, high-resolution technique for spatial data acquisition [13]. Another method for surface detection is the camera-based Structure from Motion (SfM) method [14], where maps are created from a series of pictures. The method has shown its applicability in different scales from mapping landscapes [15] as well as in measuring soil roughness using a commercial grade camera [16].

The aim of this study was to use the 2D LiDAR sensor to assess the furrow geometry and area of a trailing shoe sweep. The trailing shoe is applied to establish a soil furrow to contain the liquid slurry during a slurry application. The use of the trailing shoes is expected to lower emissions in comparison to the slurry application by trailing hoses. The former reduces the surface contact between the slurry and the surrounding atmosphere, which is recognised as a significant factor [17,18,19]. Therefore, accurate measurements of how furrow geometry varies with soil texture and soil moisture content can support predictions of when application by trailing shoe is an efficient emission reduction technique. The objectives were (i) to compare two methods for evaluating soil surface and furrow cross-sectional profiles after a trailing shoe operation using a digital LiDAR sensor and an analogue pinboard and (ii) to measure the changes in furrow geometry and the spatial variations along furrows in coarse sand and loamy sand soils with three different amounts of irrigation.

### 1.1. Background

#### 1.1.1. Manual Soil Surface Measurements

The relief meter, which was an early version of the pinboard was presented for measuring the soil roughness between cultivation and seedbed preparation [10]. The pinboards vary in size, numbers of pins, spacing between those, materials used, and method of collecting data, but the principle of the measurements is the same. The method is commonly used as it is cost effective and easy to operate. Since the early development, the pinboard method has been used to measure soil surface variations for different purposes [20,21]. Further development of the pinboard for measuring soil surface roughness and soil variations at the microscale was presented [22]. The pinboard has also been used for evaluating soil loss from quarter-drains by quantifying the changes of the cross-sectional area before and after rain events [23].

#### 1.1.2. LiDAR and Camera-Based Soil Surface Sensing

In agricultural engineering, LiDAR-based systems have been applied for various applications such as obstacle detection for autonomous field-robots [24], 3D imaging of crop development [25], and soil surface analysis [6]. The automated systems for soil surface detection have been presented since the 1980s [26]. Moreover, sensor-based systems have been applied for studying microtopography and soil roughness on different scales, from point samples [27,28] to larger areas [29] as well as for airborne applications [30]. The laser-based method performs within the accuracy of the pin displacement unit [31], which made the laser a feasible tool for researching erosion [32]. In addition, microtopography effects of rainfall has been studied using both a photogrammetric technique and a high-resolution laser scanner [33] as well as 1D distance sensors [34,35] and 2D scanners [36] have been applied for soil roughness estimations [37,38]. Besides the use of scanners, camera-based techniques have also been applied to determine soil surface roughness, from hand-held cameras [39] to modern commercial grade cameras [40]. In addition, stereo photogrammetry was also used for mapping soil surfaces [41] and estimating microtopography and soil roughness [42]. Many stationary LiDAR and camera-based systems have been presented for automated soil surface measurements. However, LiDAR was mentioned as a feasible choice for stationary use as well as for on-the-go sensing of soil roughness in the field [3].

## 2. Materials and Methods

### 2.1. Experimental Settings

**Figure 1:**
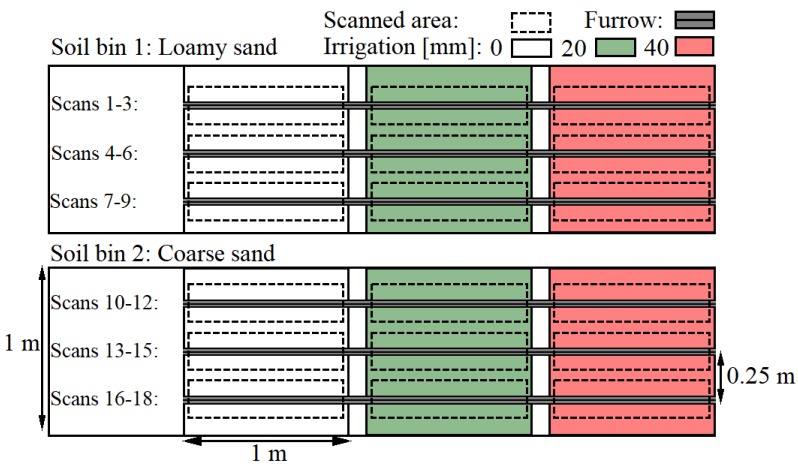
Experimental design sketch. 18 furrows were obtained and measured using the pinboard and LiDAR within six plots of two soil types and three irrigation amounts.

In order to compare two methods such as the pinboard and LiDAR, an experiment consisting of 18 trailing shoe furrows which were obtained in two semi-field soil bins at three irrigation amounts was conducted (Figure 1). For each furrow, a 3D scan and a pinboard measurement of the geometry and area were obtained.

The experiment was conducted in the semi-field facility at Aarhus University Foulum Research Center, Tjele. The semi-field facility was established in 1993 [43], consisting of three soil bins under a moveable roof that allows for controlling the precipitation. Three weeks prior to and during the experiment, the roof was kept over the studied soil bins and the soil was kept bare during the experiments. Two out of three semi-field soil bins were used in this research, coarse sand and loamy sand [44,45,46,47]. The coarse sand (Ortic Haplohumod) had 4% clay (<0.002 mm), 5% silt (0.002–0.063 mm), and 2.0% soil organic matter, while loamy sand (Typic Hapludult) was characterized by 9% clay, 24% silt, and 2.5% soil organic matter [48]. Approximately 24 h prior to the experiment, six plots (1 × 1 m) were irrigated with three different amounts of tap water: 0, 20 and 40 mm. The soil water contents were determined gravimetrically [49] using a soil core of 100 cm${}^{3}$ (Table 1). The dry bulk densities were 1.28 ± 0.09 g cm${}^{-3}$ for loamy sand and 1.39 ± 0.07 g cm${}^{-3}$ for coarse sand. Due to an unusually dry and hot period prior to the experiments, no significantly different soil water contents were obtained except for the coarse sand soils with 40 mm of irrigation compared to the non-irrigated coarse sand soils (Table 1).

**Figure 2:**
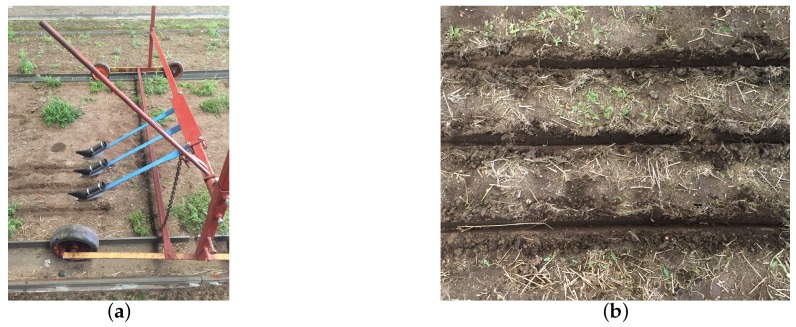
Frame with three of the Bomech trailing shoes (**a**) and three furrows in coarse sand at 40 mm irrigation (**b**).

In order to study soil displacements of the furrows, three Bomech trailing shoes (Bomech B.V., Albergen, The Netherlands) were attached to a metal frame on wheels with a distance of 25 cm between each trailing shoe (Figure 2a,b). A vertical load of 117.7 N (12.0 kg) was exerted on each trailing shoe. Three sweeps were conducted for each soil bin at a constant speed of approximately 2.0 km h${}^{-1}$. In total, 18 furrows were obtained and measured using the analogue pinboard and the digital LiDAR approaches.

### 2.2. LiDAR-Based Soil Surface Measurement Unit

**Figure 3:**
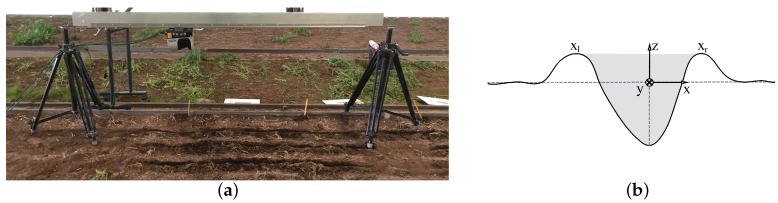
The LiDAR-based soil surface measurement unit in the semi-field facility (**a**); Sketch of the furrow geometry that includes the reference frame of the cross-sectional profile (**b**), solid line indicates the soil surface and dashed lines indicate the vertical and horizontal axis of the origin.

A soil surface measurement unit was assembled to obtain high-resolution and continuous scans of the soil surface. The 2D LiDAR sensor (SICK LMS511-20100 PRO) was chosen based on the indicated applicability for roughness measurements in the field [50]. The LiDAR operates from a minimum distance to the target at 0.70 m. According to the operating instructions, the statistical error is ±7 mm at 1–10 m distance. The LiDAR was mounted on a linear rail system (Figure 3a) that allowed to obtain the 3D scans of the soil surface by moving the scanner at an elevation of 0.75 m above the surface and parallel to the direction of the furrow while monitoring the position of the scanner. The scans were conducted with an angular resolution of 0.167${}^{\circ }$ and an angular frequency of 25 Hz, while the linear speed of the scanner ($\dot{y}$) (Figure 3b) was constant at 0.003 m s${}^{-1}$, driven by a stepper motor. The distance between two line scans along the furrow (y-direction) was 0.33 mm. Each line scan was 37 cm wide and consisted of 166 data points with a maximum distance between two points of 2.3 mm. The beam diameter increases from 13 mm at the front screen of the sensor to approximately 16.5 mm in diameter at the soil surface of this elevation using the high resolution setting of the LiDAR.

The raw sensor data was collected using the software, SOPAS Engineering Tool ver. 2018.2 (SICK, Germany). No automatic filtering was applied during data sampling. However, the data points were transformed from polar coordinates into cartesian coordinates to study the vertical distances from the scanner to the soil surface in the post-processing and to assemble the 3D scans. A low-pass Gaussian filter (σ=1.5) was applied in order to remove sensor noise before generating 3D scans using the package: *scipy.ndimage.filters.gaussian_filter* of SciPy ver. 2.0 in Python 3.6.5.

The x and z coordinates were obtained using the LiDAR sensor, whereas the y coordinates were monitored through the stepper drive (Figure 3b).

### 2.3. Furrow Cross-Sectional Geometry and Area Measurements

#### 2.3.1. LiDAR Measurements

3D scans of the furrows were obtained using LiDAR measurements and generated based on the filtered data. In order to compare the results of the 3D scanned furrows, a reference frame was introduced to each measured furrow profile. A coordinate system was located in the xz-plane of the furrow. The horizontal xy-plane was located at the elevation of the undisturbed soil. The yz-plane was intersecting with the deepest point of the furrow (Figure 3b). A *mean furrow* geometry was introduced to express 3D scans as 2D profiles and, hence, to visualise the spatial variations along furrows. The mean furrow was defined for each scan as the mean of the vertical distance measurements (z) for each value of (x) in the relative coordinate systems along the furrow (y). The mean furrow geometries were generated based on 3D scans of which the area was calculated using the package *sklearn.metrics.auc ver. 0.19.1* in Python 3.6.5 by integrating the elevations measurements (z) over the distance $x_{l}$ to $x_{r}$ using the trapezoidal rule (Figure 3b). The 2D mean furrow and standard deviations were obtained based on one scan per centimetre of the furrows.

#### 2.3.2. Pinboard Measurements

**Figure 4:**
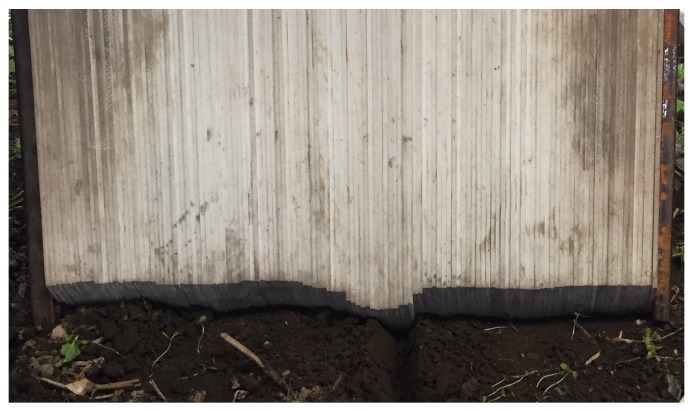
The analogue measurement using pinboard of the furrow in loamy sand at 40 mm irrigation.

The analogue measurements of the furrow geometries and areas were performed using pinboard (Figure 4). The pinboard consists of pins (width of 3 mm) that moves freely in the vertical direction. The device was operated by levelling the pins on the soil surface. The contour of the surface was then drawn on paper based on the position of the pins. Subsequent to this operation, the areas were obtained by moving a Digitizing Area-line Meter (Super PLANIX b, Tamaya Technics Inc., Tokyo, Japan) along the drawn contour of the furrow profile, hence referred to as the analogue area measurements. This procedure was done three times for each analogue cross-sectional measurement, and the mean value of these were used for further analysis. In this study, the Computer Aided Design (CAD) software, SOLIDWORKS 2018 SP1 (Dassault Systémes, Paris, France) was applied to digitalise the drawn pinboard-based contours.

## 3. Results and Discussion

### 3.1. 2D and 3D Soil Surface Profiles

**Figure 5:**
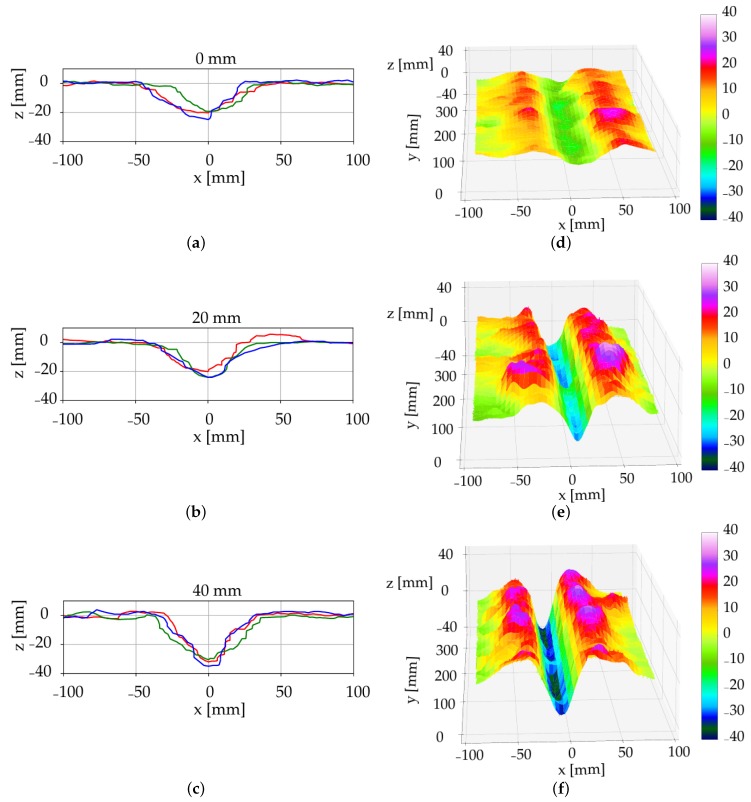
The cross-sectional measurements in coarse sand after irrigation of 0 mm(**a**,**d**), 20 mm (**b**,**e**), and 40 mm (**c**,**f**). Pinboard measurements (**a**–**c**), where three repetitions are shown as red, blue and green lines and LiDAR measurements (**d**–**f**), where the colourbar represent elevation (z) in mm.

**Figure 6:**
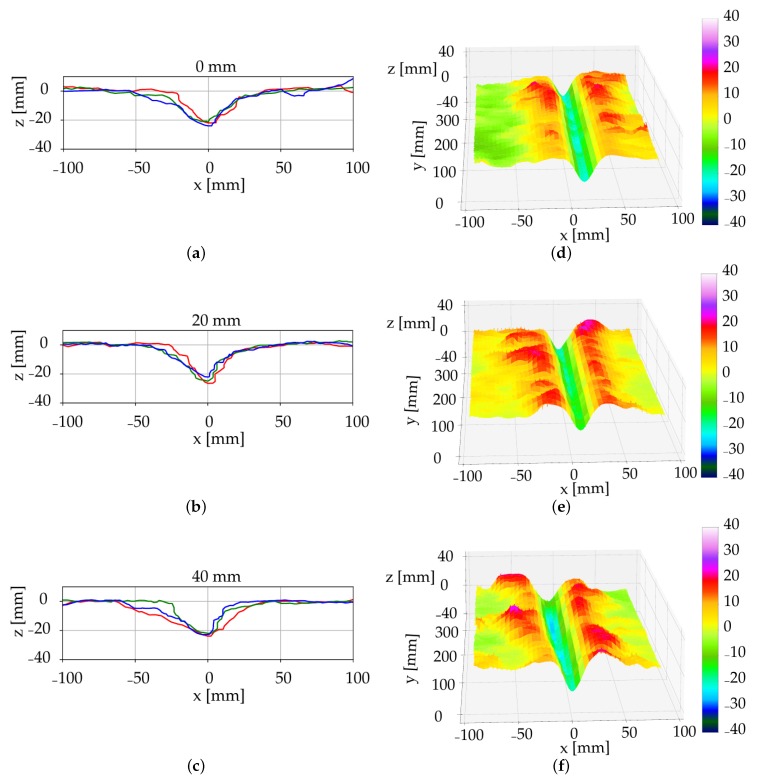
The cross-sectional measurements in loamy sand after irrigation of 0 mm (**a**,**d**), 20 mm (**b**,**e**), and 40 mm (**c**,**f**). Pinboard measurements (**a**–**c**), where three repetitions are shown as red, blue and green lines and LiDAR measurements (**d**–**f**), the colourbar represent elevation (z) in mm.

The 3D scans allowed to evaluate furrow geometries as a result of different irrigation amounts as well as to estimate the variation along furrows within a larger area. The LiDAR gave a more representative description of the furrow compared to analogue measurements as only a limited number of manual measurements can be conducted along the furrow due to the time-consuming process [6]. The cross-sectional geometry, such as width, depth and shape, varied in the two soil types, where the loamy sand plots with no irrigation were characterized with a more well-defined edge and a narrower furrow-width compared to the non-irrigated coarse sand plots (Figure 5 and Figure 6). The furrow geometry in the coarse sand was affected by the increased soil water content. However, in the loamy sand it was not possible to conclude on furrow geometry changes as a factor of soil water content due to the fact that the latter was not significantly different (Table 1). Although, the experiments were performed in semi-field conditions under controlled irrigation, the LiDAR will provide similar results under field conditions. The soil surface in the semi-field is comparable to soil surface conditions in fields, however, a limitation of the LiDAR is the need for bare soil to obtain an uninterrupted line of sight between the sensor and the soil surface.

The furrow profiles of the two soil types under the three irrigation amounts were illustrated in Figure 5 and Figure 6. The subfigures, (a–c) present the 2D profiles of the pinboard measurements, three repetitions each, digitalised using CAD software. The subfigures (d–f) present a subset of the furrow 3D scans and was generated based on the stepper position and LiDAR measurements. In the coarse sand plots, a visual effect of the irrigation was observed by leaving a deeper furrow compared to the non-irrigated soil (Figure 5). This indicates that the resulting furrow geometry was dependent on the water content. Hence, water content and soil type should be considered when the trailing shoe is used for the liquid slurry application.

**Figure 7:**
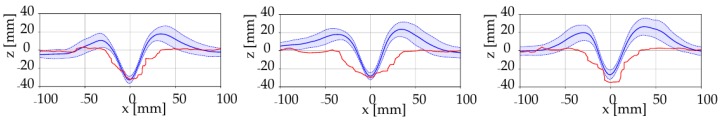
Mean profiles (solid blue lines), standard deviation on the elevation measurement (light blue area), analogue pinboard measurement (red line). Results are shown for all repetitions in coarse sand at 40 mm irrigation.

The mean furrows and the standard deviations of the elevation measurements in the direction of the furrows were determined as the spatial variation along the furrows was observed. Using the mean furrow, it was possible to present 3D scans as 2D cross-sectional plots, which allowed to compare the digital mean furrow plots with the analogue pinboard measurements of the furrow (Figure 7) as the common reference frame was applied (Figure 3b). The location of the common reference frame was a feasible choice for the soil bin study. However, in a field setting, another choice of reference frame could be introduced, e.g., the deepest point of a furrow.

The ability to visualize and take into account the variation along the furrow is needed for research and development purposes related to soil and tillage tools [51]. The magnitude of the standard deviation provides an insight into the variation of the furrow geometry. By evaluating the 3D scans, the three-dimensional soil displacement become available in the assessment of tillage tools [52]. The pinboard has previously been applied for comparing results of simulations-based and measured soil-tool interactions [48]. By considering the variations along the furrow, it is possible to include a physics-based error tolerance when validating numerical models of corresponding soil-tool interactions [53].

The non-contact and automated approach of the LiDAR ensures consistent test results independent from soil conditions, which is an advantage for research purposes. The LiDAR has previously shown applicability in a field setting performing on-the-go measurements of soil surface [50]. This indicates that this technology can be further developed for applications in a field setting as a method for increasing the site-specific treatments based on LiDAR measurements. With respect to the slurry application using trailing shoes, on-board measurements of the furrow geometry and furrow area could act as an input for controlling the pressure on the trailing shoe to obtain the desired furrow area, which is shown to be dependent on the soil type and water content.

### 3.2. Furrow Area Measurements

**Figure 8:**
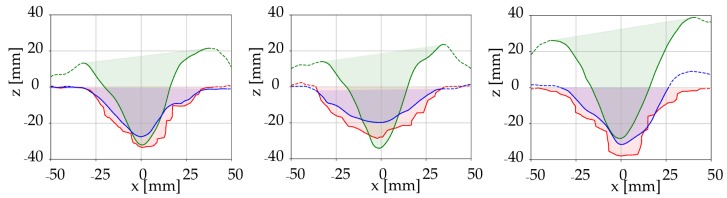
Cross-sectional geometries in coarse sand at irrigation amount of 40 mm. Line scans prior to the pinboard measurement (green line), line scan subsequent to the pinboard measurement (blue), and analogue pinboard measurement (red).

The cross-sectional area of the furrow is important due to the application of the trailing shoe, namely to establish a furrow in which the liquid slurry is contained. Furthermore, high variations in the reported effect of trailing shoe on emission reduction were observed [54,55,56,57]. The inconsistencies in reported emission reduction by trailing shoe are often attributed to the soil properties, particularly, soil moisture content [17,56], as this affects the furrow cross-sectional area. Hereby, accurate and consistent methods for assessing the cross-sectional area under different conditions are important as well as being able to correlate the volume of the furrow with the volume of the slurry. The cross-sectional area was a subject for the comparison between the digital and analogue soil surface measurement techniques. Other parameters as width and depth could in a similar way have been considered as the measure for comparison. The cross-sectional areas were measured in every soil plot (Figure 1) in order to determine the change in area due to various irrigation amounts in the two soils. The digital cross-sectional areas were determined using the mean furrow geometry of the 3D scans (Figure 7). A comparison between the analogue profiles and the closest line scan as well as an example of the areas applied for the pinboard measurements are shown in Figure 8.

**Figure 9:**
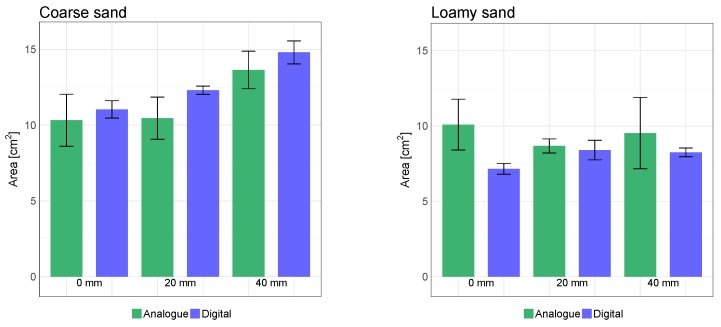
Cross-sectional area measurements based on pinboard (Analogue) and LiDAR scans (Digital) in the two soils.

The analogue cross-sectional area measurements and the LiDAR-based mean furrow areas were shown in Figure 9. The values are based on the mean of the three repetitions for each plot, i.e., combination of soil type and irrigation amount. As an example, for the coarse sand at 40 mm irrigation, the analogue area was calculated as the mean of the area above the red curves of Figure 7, whereas the digital area corresponds to the average area above the three solid blue curves.

There was no significant difference in the areas measured by the digital and analogue methods, except for the non-irrigated coarse sand and coarse sand irrigated at 40 mm (Tukey’s HSD, *p* < 0.05). However, by comparing the areas of the digital and analogue methods in the coarse sand, it was found that the analogue pinboard predicted lower than the estimations of the digital approach by 7%, 15% and 8% for the 0, 20, and 40 mm irrigation amounts. In the loamy sand plots, all the analogue measurements provided larger cross-sectional areas compared to the digital measurements of 41%, 3% and 16%, respectively. This inconsistency indicated that the results of the analogue measurements was affected by the process of first measuring the surface contour using the pinboard and then calculating the area. By comparing the magnitude of the standard deviations of the analogue area measurements and the standard deviation based on the scanned area, it was found that the standard deviation was lower when LiDAR scans were used, compared to the analogue approach. Hence, the scanner provided more consistent results than the analogue method due to the non-contact and automated process. It was also expected as that the number of data points collected for 3D scans and the mean furrows was higher than the data points collected for the pinboard measurements. By comparing the digital cross-sectional area estimations for the dry coarse sand soil with the irrigated coarse sand of 20 and 40 mm, an increase in furrow cross-sectional area was obtained by 11% and 34%. For the loamy sand, the cross-sectional area increased 17% and 15% by irrigation of 20 and 40 mm. Nevertheless, more experiments under different soil conditions and irrigation amounts are needed to fully evaluate these effects.

### 3.3. Impact Analysis of Pinboard Measurement

**Figure 10:**
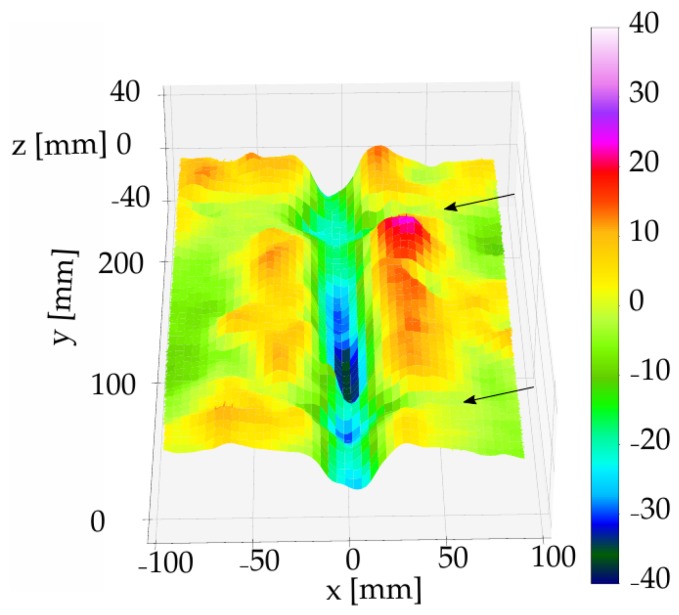
3D scan subsequent to a pinboard measurement. The arrows indicate the location of two pinboard measurements. Experiments conducted in coarse sand, irrigation 40 mm.

The disturbance of the surface in loose soils during the application of the pinboard [58] was studied by comparing the digital 2D mean furrow geometries and the analogue pinboard measurements. Differences in the furrow geometry between the analogue and digital measurements were observed in the coarse sand plots as a result of the analogue measurement. The impacts were measured by scanning the surface prior to and after the pinboard measurements. In Figure 10, a 3D scan of the soil after the analogue measurement is shown. The positions where the pinboard measurements were taken are marked with arrows (Figure 10). 2D line scans of the furrows in coarse sand with 40 mm irrigation were compared to the corresponding pinboard measurement.

It was observed that the analogue measurements did not accurately capture the geometry of the furrow. Particularly, the features of the furrow for values z > 0, using the notation of Figure 3b, were not captured. Furthermore, it was observed that the analogue measurements provide a wider representation of the furrow cross-sectional geometry compared to the scan conducted before the pinboard measurements (Figure 8).

By the means of the LiDAR, an assessment of the pinboard performance was accessible and an issue of the pinboard was identified, namely, the pinboard approach was challenged in capturing small cross-sectional geometries in loose sand due to disturbance of the soil. This issue has previously been reported as an uncertainty when using the pin-based methods in sandy soil [31].

## 4. Conclusions

Two methods were compared to evaluate the soil surface and furrow cross-sectional area after a trailing shoe sweep in two soils. A 2D mean profile of the furrow was constructed to compare the digital LiDAR-based results with the results of the analogue pinboard. From the experiments it was concluded:

(i) The geometric variations in the direction of the furrow were observed by increasing the resolution from 2D pinboard measurements to 3D scans.
(ii) The results indicated the importance of applying a non-contact method for accurate soil surface measurements in loose soils. The analogue furrow areas were below the LiDAR-based areas in coarse sand by up to 15% and for loamy sand, the analogue areas were above the LiDAR-based areas by up to 41%.
(iii) An increase in the cross-sectional areas was measured using the LiDAR-based method, in coarse sand the area increased by 11% and 34% for the 20 and 40 mm irrigation compared to the dry coarse sand. In the loamy sand, the cross-sectional area of the furrow increased 17% and 15% by irrigation of 20 and 40 mm. However, more experiments are needed to fully evaluate these effects.

## Figures and Tables

**Table 1:** Soil water content [g/g] (n = 3) with the standard deviation after three irrigation amounts such as 0, 20, and 40 mm were measured in the topsoils (0–10 cm). The letters indicate significance based on the Tukey’s HDS test, *p* ≤ 0.05.

Irrigation Level, mm	Loamy Sand		Coarse Sand	
0	0.13 (0.02)	a	0.06 (0.00)	a
20	0.16 (0.00)	a	0.08 (0.01)	ab
40	0.15 (0.03)	a	0.10 (0.02)	b
